# Otorrhœa

**Published:** 1847-09

**Authors:** James Bryan

**Affiliations:** Lecturer on Surgery, formerly Professor of Surgery and Medical Jurisprudence in the “Academy of Medicine,” Castleton, Vermont, &c., &c.


### MEDICAL EXAMINER,

AND

### RECORD OF MEDICAL SCIENCE.

NEW SERIES.—No. XXXIII.—SEPTEMBER, 1847.

### ORIGINAL COMMUNICATIONS.

*Otorrhœa.* By JAMES BRYAN, M. D., Lecturer on Surgery, formerly Professor of Surgery and Medical Jurisprudence in the "Academy of Medicine," Castleton, Vermont, &c., &c.

The subject of otorrhœa, or a discharge, sometimes purulent, from one or both ears, is one of considerable importance, both to the patient and to the medical practitioner. The disease is very common, particularly in infancy and childhood. From being at first a discharge from the lining membrane of the meatus auditorius externus, affording relief to some other local irritation, and acting on the principle of *counter-irritation*, it becomes chronic, and is itself a disease.

In teething, ophthalmia, and other inflammations, nature very commonly establishes this discharge, which undoubtedly relieves these diseases.

From this fact, which is matter of common observation, both among the profession and the people, together with the aphorism which says, "Suppression of discharges from the ears, induces diseases of the brain," it is an every day affair to see otorrhœas entirely disregarded, and no means whatever taken to heal them.

So general is this, that individuals reach the period of puberty with this affection in one or both ears. The evil consequences of such continued disease, with a regular purulent discharge for years, (in one case lately under my care of thirty-two years) may be easily conceived. The lining membrane of the meatus becomes thickened; the membrana tympani is destroyed; the bones of the ear are loosened and discharged, the inflammation, as I have seen in more cases than one, attacks the mastoid cells, and forms fistulæ opening externally.

In some cases the progress of destruction is more summary, and the disease, passing through the delicate organs of hearing, attacks the membranes and even the substance of the brain, producing *then*, and not till then, "convulsions" and death.

The latter termination, though by no means so frequent as might be imagined from the proximity of the disease to the cerebral organs, is yet sufficiently common to make the subject a matter of grave consideration. At the same time it must be borne in mind, that this *introcession* is liable to take place at any time during the existence of a chronic otorrhœa; and particularly is it the case on exposure to a cold and damp atmosphere, or to any of the causes of colds or inflammations.

The evils, then, of the prolongation of this disease, may be arranged as follows:

First. It is inconvenient, the fluids not unfrequently being very offensive.

Secondly. A permanently diseased condition of the meatus auditorius is produced—or, the bones being attacked, we may have ulceration or suppuration established in the cellular structure of the mastoid process, which may continue for a long time.

Thirdly. The destruction or ulceration, or what is *very* common, the thickening of the *membrana tympani;* the latter result being, not unfrequently, induced in a few days from an otorrhœa. It is well known, in fact, that a very large proportion of individuals, who are supposed to possess good hearing, have partial deafness in one ear at least, and sometimes in both, from this cause.

The ulcerative process takes place rapidly in the delicate diaphanous membrane of the tympanum; and although it has been shown by some good writers that the perforations induced by ulceration, may, by proper treatment, be healed; yet where there is no treatment, and the ulcerations heal up in the ordinary course of the disease, it is fair to infer, that the perforations remain for life. I recollect when a boy, in company with others, boys and men, a common amusement among them was to blow smoke from the segars they were using, *through their ears*. And a considerable per centage of these individuals in a country village could perform this feat. The destruction of the membrane itself, however, I suspect, is the most usual termination of the disease. After this it not unfrequently gets well itself, and the discharges cease.

Fourthly. The ossicula auris are loosened and discharged, and the fenestra ovalis is exposed to the contact of atmospheric air. The apparatus so important to good audition—viz., the membrana tympani and bones of the ear—being lost, the function must necessarily be very much impaired; in some cases the Eustachian tubes close, and in this way the external and middle ears are entirely impaired.

Fifthly. The possible, nay probable, occurrence of a transfer of the inflammatory action to the brain or its membranes, by metastasis or *continuous* inflammation, is an argument of the highest importance in this discussion.

Sixthly. The partial or complete loss of the function invariably accompanying the disease, should be inducement enough for us to investigate the propriety of allowing these discharges to continue, without medical interference.

The arguments in favour of non-interference may be summed up in a few words:

First. It is a diverticulum of nature which it is dangerous to interfere with.

Secondly. It very frequently (the object of nature being obtained) heals up itself, leaving no great derangement of the organ.

In reference to the first, we would remark that although a diverticulum, (or source of counter-irritation,) and in some cases protecting important organs, yet the seat of the discharge is itself an important organ, and no man would select *that* as the place to establish counter-irritation in the case of disease in any other organ, especially as it is well known that a purulent discharge from behind the ear, the back of the neck, or the arm, would be productive of all the good effects claimed for a discharge from this locality.

We might with the same propriety establish inflammation in the eye ,for the relief of cerebral, dental, or aural diseases, and in this way impair or destroy the function of that organ, whose structure is so delicate, and yet no *more* delicate than that of the ear.

But, say the advocates of the do-nothing-practice, what are you to do? "Suppression of discharges from the ears induces diseases of the brain," and daily experience shows this to be a fact. Granted. Cannot the disease be *transferred* from the organ of hearing to some other less important part, and there maintained to an indefinite period without the danger of producing any evil consequences? Certainly it can; and if not *cured* in the sense meant by the above aphorism, yet its seat of action may be changed, and ultimately, by judicious attention to the case, allowed to heal up entirely, as is the practice in almost any other instance, where for a time an issue or purulent discharge is desirable. There is nothing peculiar in the structure of the ear, that gives it any superiority as a seat of inflammatory action, to relieve other parts in a diseased or tending to a diseased condition. So far from this being the case, we are of the opinion that the skilful physician is not justified in allowing this discharge to continue a *single day* in *any* case, where he, by slight irritation behind the ear, or the back of the neck, or other parts, is enabled to arrest it, but on the contrary he should be as anxious to stop it as he is to arrest a purulent ophthalmia or other inflammatory affection of the eyes.

We cannot agree with Dr. Bonnafont, as quoted in No. 5, of "Ranking's Abstract," that "chronic mucous discharges from the ear are always owing to ulceration of the parietes of the external auditory canal, or of the membrane of the tympanum." For it is well known that polypous and other growths produce the same discharges, and great care should be taken in the examination of the auditory canal, with the speculum in a *sun light*, lest these productions escape our notice. They should, in fact, be always *suspected* to exist in cases of chronic otorrhea. That "these discharges, generally very easily treated in their acute stage, frequently become very obstinate, and terminate in disorders which almost always involve a more or less serious degree of deafness, and sometimes the death of the individual," are facts which should influence the profession, we are fully convinced.

We have found, in reference to the treatment, that in the early stages, in uncomplicated cases, the production of moderate irritation behind the ear—with careful syringing, first with tepid water, to wash out the secretions, followed by a solution of the plumb. acetat. in the proportion of from two to six grains to the ounce of distilled water—all that was necessary. A small half-moon blister, or a drop or two of croton oil, or the latter used in the form of unguent, are means usually resorted to, to establish this irritation. When complicated, however, with general or local disease, acting as concomitant, and sometimes, as the cause of the otorrhea, the treatment must necessarily be varied to suit the case. Scrofula, particularly in children, is a very common condition connected with the disease, and requires the usual constitutional treatment, in addition to the local applications. General debility, gastric irritation, papular eruptions, spreading erysipelatous and other cutaneous diseases, induce otorrhœas, and should be treated accordingly. Cases of simple acute otorrhœa, induced by cold or any of the common causes, are treated so successfully by the above, or similar means, that the publication of cases would scarcely be deemed advisable. Of the chronic forms, however, which are considered more difficult to cure, and many of which are absolutely incurable, the following cases may be found instructive.

Miss B., aged fourteen years, light complexion, rather full habit, has had a discharge from both ears for about a year; hearing distance with a watch in left ear, one foot; in the right the watch could be heard indistinctly when placed in contact with the ear. The treatment consisted in washing out the canal well, with tepid water, and then throwing in the aqueous solution of the acetate of lead; four grains to the ounce; and the application of blisters behind the ears. The hearing began to improve immediately, so that at the end of a week the discharge from the left ear had ceased, and the hearing distance increased to six feet. The hearing distance in the right ear was restored to four inches, the discharge continuing. At the end of four weeks the improvement in the right ear became stationary, at ten inches hearing distance; the discharges though small, remained.

On close inspection it was found that a moderate sized polypous growth had been overlooked, on account of the tumefaction in the first examination, in the lower part of the middle third of the auditory passage. This was carefully excised, and the blister-surface restored behind the ear,—with the use of the aqua plumbi. In four days the discharge had nearly ceased; the hearing distance advanced to two feet; and in ten days the discharge of both ears no longer existed; the hearing distance was respectively six and ten feet.

Second case, August 1846.—Mr. B., an artist, dark complexion, aged thirty-four years, has had a discharge from both ears ever since he was two years old. The hearing distance, as indicated by my watch was, in the left ear two inches, in the right ear four inches.

On examination with the speculum, the membrane of the tympanum of *the left ear* was entirely destroyed, together with the bones of the middle ear. When air was driven by the patient through the Eustachian tube, the fluids were *seen* to bubble and heard to rattle in the ear.

The discharge from this ear was not great, and chiefly of a thin watery character.

The membrane of the tympanum of the right ear was destroyed to about two-thirds of its extent, and the bones of the ear were exposed; the handle of the malleus projected into the portion of the membrane which remained, which was also thickened and ulcerated, discharging with the surrounding surface a large quantity of fœtid purulent fluid. The Eustachian tube was here also pervious, as shown by the air press and catheter, and by voluntary sufflation.

Half moon blisters were applied behind the ears, and an ear lotion of four grains of the acetate of lead, to an ounce of water was directed, to be applied after carefully syringing the ears out with clean tepid water three times a day.

This treatment was continued with the use of a mild astringent gargle, (the throat being slightly inflamed) for three days, when my patient left the city for his residence, some eighty miles distant, in New Jersey, to return at the end of a week or ten days.

On the 31st of August he again called upon me, and I found the blistered surface healed. The discharge from the left ear had ceased, and the internal surface was dry and shining. The hearing distance had increased to four inches.

The discharge from the right ear had diminished, but the process of destruction was evidently still going on. The hearing distance had however improved to about five inches. The blisters were re-applied with directions to add a small quantity of the ceratum cantharidum to the ordinary dressings, in order to keep up the irritation and discharge. The strength of the lotion was increased to six grains of the acetate of lead to the ounce, and the fluid extract of sarsaparilla, as prepared by Maris & Co., of this city, was directed to be taken in teaspoonful doses twice a day. The gargle to be continued—exercise in moderation to be taken, and to avoid coffee, and most of the unnecessary stimulants. On the 2d of September he left for his home, the hearing distance having increased to six inches in the left, and seven inches in the right ear. The discharge from the right ear almost nothing.

Sept. 17th. The hearing continues in the improved condition; the discharge from right ear still to a small extent. The sulphate of copper in solution was directed as a lotion. The etherial vapour was introduced a few times through the Eustachian tubes, with some small improvement in the sensibility of the nerves of audition. The ears were directed to be kept warm, and protected from dust and changes of temperature, by the use of wool in the auditory canal.

On the 19th patient left the city for his home, with the hearing improved in the right ear to ten inches, the discharge having *entirely ceased*; and in his left, to something more. One year nearly has passed away and the improvement continues.

Other cases might be taken from our case-book, but we did not design to make a long article; "sapienti verbum sat."

